# Supplementary material for: Troponin in COVID-19: To Measure or Not to Measure? Insights from a Prospective Cohort Study
Source: J Clin Med. 2022 Oct 9;11(19):5951. doi: 10.3390/jcm11195951 (PMC9570932; doi:10.3390/jcm11195951)
Supplement: Supplementary file 1 [file jcm-11-05951-s001.zip › jcm-1903588-supplementary.pdf]

**Supplementary Material**

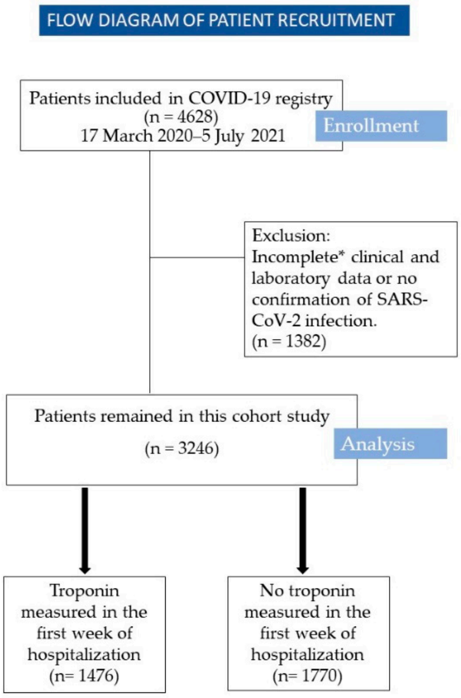


**Figure S1.** Flow diagram of patient recruitment

*Patients were excluded from the analysis due to impossibility of clinical data collection in some centers during turbulent periods of the pandemic or no confirmation of SARS-CoV2 infection.

**Table S1.** Troponin assays.

| Troponin assay | 99th percentile URL (ng/L)* | | |
|---|---|---|---|
| | Overall | Male | Female |
| Vitros Fusion 5.1 Troponin I hs | 11 | 13 | 9 |
| BioMérieux VIDAS 3 Troponin I hs | 19 | 25 | 11 |
| ADVIA Centaur Troponin I hs | 47 | 57 | 37 |
| XT 76000 Troponin I hs | 11 | 13 | 9 |
| Vitros 5600 Troponin hs | 11 | 13 | 9 |
| Roche cTnT-hs STAT on cobas e 601, 602, E170; commercial | 14 | 16 | 9 |
| Abbott / ARCHITECT I systems / hs Troponin-I | 26.2 | 34.2 | 15.6 |

URL, upper reference limit. *According to manufacturer.

**Table S2.** Baseline characteristics of patients according to serum troponin levels.

| Characteristics | All patients (n=3246) | Troponin levels (URL) ≤ 1x (n=1123; 34.6%) | > 1 to 3x (n=127; 3.9%) | > 3 to 10x (n=87; 2.7%) | > 10x (n=139; 4.3%) | Not measured (n=1770; 54.5%) | χ2 test P-value |
|---|---|---|---|---|---|---|---|
| **Sex, No (%)** | | | | | | | |
| Male | 1935 (59.6) | 667 (34.5) | 85 (4.4) | 59 (3.0) | 84 (4.3) | 1040 (53.7) | 0.210 |
| Female | 1311 (40.4) | 456 (34.8) | 42 (3.2) | 28 (2.1) | 55 (4.2) | 730 (55.7) | |
| **Age, No (%)** | | | | | | | |
| < 40 years | 603 (18.6) | 235 (39.0) | 5 (0.8) | 6 (1.0) | 8 (1.3) | 349 (57.9) | < 0.001 |
| 40 to 59 years | 1357 (41.8) | 524 (38.6) | 30 (2.2) | 20 (1.5) | 35 (2.6) | 748 (55.1) | |
| 60 to 69 years | 541 (16.7) | 174 (32.2) | 29 (5.4) | 21 (3.9) | 36 (6.7) | 281 (51.9) | |
| ≥70 years | 745 (23.0) | 190 (25.5) | 63 (8.5) | 40 (5.4) | 60 (8.1) | 392 (52.6) | |
| **$O_2$ Sat, No (%)** | | | | | | | |
| ≥ 94% | 2327 (71.7) | 832 (35.8) | 84 (3.6) | 45 (1.9) | 94 (4.0) | 1272 (54.7) | < 0.001 |
| ≤ 93% | 919 (28.3) | 291 (31.7) | 43 (4.7) | 42 (4.6) | 45 (4.9) | 498 (54.2) | |
| **Diabetes, No (%)** | | | | | | | |
| No | 2434 (75) | 869 (35.7) | 84 (3.5) | 55 (2.3) | 95 (3.9) | 1331 (54.7) | 0.001 |
| Yes | 812 (25) | 254 (31.3) | 43 (5.3) | 32 (3.9) | 44 (5.4) | 439 (54.1) | |
| **Hipertension, No (%)** | | | | | | | |
| No | 1403 (43.2) | 526 (37.5) | 27 (1.9) | 20 (1.4) | 41 (2.9) | 789 (56.2) | < 0.001 |
| Yes | 1843 (56.8) | 597 (32.4) | 100 (5.4) | 67 (3.6) | 98 (5.3) | 981 (53.2) | |
| **Heart Failure, No (%)** | | | | | | | |
| No | 3145 (96.9) | 1103 (35.1) | 113 (3.6) | 81 (2.6) | 131 (4.2) | 1717 (54.6) | < 0.001 |
| Yes | 101 (3.1) | 20 (19.8) | 14 (13.9) | 6 (5.9) | 8 (7.9) | 53 (52.5) | |
| **Coronary disease, No (%)** | | | | | | | |
| No | 3029 (93.3) | 1060 (35.0) | 103 (3.4) | 76 (2.5) | 124 (4.1) | 1666 (55.0) | < 0.001 |
| Yes | 217 (6.7) | 63 (29.0) | 24 (11.)1 | 11 (5.1) | 15 (6.9) | 104 (47.9) | |
| **Chronic heart disease, No (%)** | | | | | | | |
| No | 3079 (94.9) | 1075 (34.9) | 111 (3.6) | 76 (2.5) | 122 (4.0) | 1695 (55.1) | < 0.001 |
| Yes | 167 (5.1) | 48 (28.7) | 16 (9.6) | 11 (6.6) | 17 (10.2) | 75 (44.9) | |
| **Chronic kidney disease, No (%)** | | | | | | | |
| No | 3094 (95.3) | 1094 (35.4) | 118 (3.8) | 77 (2.5) | 129 (4.2) | 1676 (54.2) | < 0.001 |
| Yes | 152 (4.7) | 29 (19.1) | 9 (5.9) | 10 (6.6) | 10 (6.6) | 94 (61.8) | |

URL, upper reference limit, $O_2$ sat, oxygen saturation.

**Table S3.** In-hospital mortality according to troponin level in the first week.

| Troponin | All patients n (%) | In-hospital mortality No n (%) | Yes n (%) | CI95% | χ² test *p*-Value |
|---|---|---|---|---|---|
| ≤ 1 x URL (no injury) | 1123 (100) | 1061 (94.5) | 62 (5.5) | 4.3 - 7.0 | < 0.001 |
| >1x URL (injury) | 353 (100) | 273 (77.3) | 80 (22.7) | 18.5– 27.3 | |
| > 1 to 3x URL | 127 (100) | 110 (86.6) | 17 (13.4) | 8.3 - 20.2 | |
| > 3 to 10x URL | 87 (100) | 67 (77.0) | 20 (23.0) | 15.1 - 32.7 | < 0.001 |
| > 10x URL | 139 (100) | 96 (69.1) | 43 (30.9) | 23.7 - 39.0 | |
| Measured | 1476 (100) | 1334 (90.4) | 142 (9.6) | 8.2–11.2 | 0.04 |
| Not measured | 1770 (100) | 1559 (88.1) | 211 (11.9) | 10.5 - 13.5 | |
| All patients | 3246 (100) | 2893 (89.1) | 353 (10.9) | 9.8 - 12.0 | |

CI, confidence interval; URL, upper reference limit.

**Table S4.** In-hospital mortality by complications (*n* = 3246).

| Complications | In-hospital mortality | RR | $c^2$ test P-value |
|---|---|---|---|
| **Thromboembolic phenomena** | | | |
| No | 10.3% | 1 | < 0,001 |
| Yes | 25.8% | 2.5 | |
| **Stroke or TIA** | | | |
| No | 10.7% | 1 | 0.017 |
| Yes | 18.5% | 1.7 | |
| **Myopericarditis** | | | |
| No | 10.7% | 1 | 0.003 |
| Yes | 24.4% | 2.3 | |
| **Myocardial ischemia*** | | | |
| No | 10.4% | 1 | < 0,001 |
| Yes | 33.3% | 3.2 | |
| **Acute renal failure** | | | |
| No | 4.7% | 1 | < 0,001 |
| Yes | 57.6% | 12.3 | |
| **Sepsis or septic shock** | | | |
| No | 5.8% | 1 | < 0,001 |
| Yes | 54.0% | 9.3 | |
| **Invasive ventilation** | | | |
| No | 1.4% | 1 | < 0,001 |
| Yes | 51.0% | 36.4 | |

*Acute myocardial infarction, myocardial ischemia, or coronary intervention.
RR, relative risk; TIA, transient ischemic attack.

**Table S5.** Complications according to troponin measurement (*n* = 3246).

| Complications | Incidence | | | | $c^2$ test p-value |
|---|---|---|---|---|---|
| | Total (n=3246) | | Troponin | | |
| | n | (%) | Measured n (%) | Not measured n (%) | |
| Thromboembolic phenomena | 120 | (3.7) | 43 (2.9) | 77 (4.4) | 0.31 |
| Stroke or TIA | 92 | (2.8) | 45 (3.0) | 47 (2.7) | 0.501 |
| Myopericarditis | 45 | (1.4) | 30 (2.0) | 15 (0.8) | 0.004 |
| Heart failure | 82 | (2.5) | 39 (2.6) | 44 (2.5) | 0.873 |
| Myocardial ischemia | 72 | (2.2) | 48 (3.3) | 24 (1.4) | < 0.001 |
| Sepsis or septic shock | 339 | (10.4) | 175 (11.8) | 165 (9.3) | 0.022 |
| Acute renal failure | 380 | (11.7) | 160 (10.8) | 220 (12.4) | 0.161 |
| Invasive ventilatory support | 621 | (19.1) | 250 (16.9) | 371 (21.0) | 0.004 |

TIA, transient ischemic attack.
